# Strategy Construction to Improve the Thermal Resistance of Polyimide-Matrix Composites Based on Fiber–Resin Compatibility

**DOI:** 10.3390/ma18245685

**Published:** 2025-12-18

**Authors:** Yu Xing, Hongjiang Ni, Daijun Zhang, Jun Li, Xiangbao Chen

**Affiliations:** National Key Laboratory of Advanced Composites, AECC Beijing Institute of Aeronautical Materials, Beijing 100095, China; xingyu5715@163.com (Y.X.); 15810534483@139.com (D.Z.); lijun19860619@126.com (J.L.); xiangbao.chen@biam.ac.cn (X.C.)

**Keywords:** polyimide composites, carbon-fiber sizing, nonlinear detriment effect, thermal resistance

## Abstract

Carbon-fiber-reinforced thermoset polyimide composites have found wide applications in various aerospace areas. In this paper, the influence of carbon-fiber sizing on the thermal properties of polyimide composites was studied. Nonlinear detriment of the epoxy sizing was found to affect the composite’s thermal resistance. Furtherly, the mechanism, possibly responsible for the nonlinear detrimental effect of the epoxy sizing, was investigated through curing kinetics analysis and chemical structure characterization. Thermal curing activation energy change was found, possibly arising from the insertion of a flexible segment into the polyimide chain by epoxy–amine reaction. Based on the proposed mechanism, a strategy to manipulate the thermal resistance was established and verified. By the precuring of the carbon-fiber sizing, the polyimide composite exhibited obvious thermal resistance improvement, indicated by an enhancement of the glass transition temperature by 20 °C, and a weight-loss reduction under thermal aging at 400 °C by 25%. Simultaneously, a good fiber-matrix interface was maintained. This strategy provides a new route to enhance the thermal properties of polyimide composites from the viewpoint of carbon-fiber-matrix compatibility.

## 1. Introduction

Polymer-matrix composites, noted for their high specific strength and specific modulus, are widely employed in aerospace engines to achieve substantial weight reduction and to expand structural design flexibility, thereby enhancing overall engine performance. The integration of advanced resin-based composites has progressively become a key indicator of technological advancement in aerospace structural development [1,2,3,4]. Among these, polyimide-based composites exhibit excellent high-temperature resistance, fatigue durability, and corrosion resistance, making them ideal for high-temperature components located in the cold sections of aircraft engines [5,6,7]. For example, polyimide composites have been applied in Ultra-high tip speed blades, F404 outer duct, F101 DFE inner duct, and PW1120/PW1130 external nozzle flaps [8,9,10,11]. Extensive practical applications have demonstrated that polyimide composites are outstanding engineering materials.

With the rapid advancement of the aviation industry, increasing the turbine inlet temperature has become an essential requirement for the development of next-generation aviation engines [12,13,14]. This trend has imposed increasingly stringent demands on the thermal resistance of materials used in the cold-section components of engines. Polyimide-based composites, regarded as ideal candidate materials, have attracted significant research interest due to their potential for enhanced high-temperature performance. The thermal resistance of these composites primarily depends on the polyimide resin matrix. In general, the higher the thermal resistance of the resin matrix, the better the overall thermal performance of the composite. Over the years, various structural systems of polyimide resins have been developed, including non-reactive end-capping, norbornene anhydride (NA)-terminated, and phenylethynyl phthalic anhydride (PEPA)-terminated, with thermal resistance levels exceeding 370 °C [15,16,17,18,19,20,21,22,23,24,25,26]. To further improve their heat resistance, recent studies have explored organic-inorganic hybridization as a means to enhance thermal performance beyond conventional organic structural modifications. Strategies include incorporating cage-like silsesquioxanes (POSS), siloxanes, and carboboranes into the molecular architecture [27,28,29,30,31,32]. One example is P^2^SI 900HT, a commercially available inorganic-organic hybrid polyimide resin, which exhibits a glass transition temperature of 489 °C (tanδ) in its pure resin form. This system demonstrates outstanding toughness, dielectric properties, and low toxicity, while maintaining mechanical integrity even after exposure to 704 °C [33,34]. Although hybridization significantly improves thermal aging stability, it also introduces challenges such as reduced interfacial adhesion, increased manufacturing costs, and more complex processing.

Sizing agents play a crucial role in composite materials, serving functions such as bundling carbon fibers and improving their surface activity and wettability [35,36]. They act as a bridge between the carbon fibers and the resin matrix at the interface of the composite, controlling load and temperature transfer within the material. By optimizing molecular structure and undergoing chemical modifications, sizing agents enhance the physical and chemical bonding at the composite interface, significantly improving interfacial properties [37,38,39]. In terms of temperature resistance, sizing agents also have a considerable impact. When composites are exposed to high-temperature environments, insufficient temperature resistance in sizing agents can lead to the exposure of sizing at the interface, forming erosion points that accelerate material degradation and reduce durability under high temperatures [40,41]. Currently, epoxy-based sizing agents are widely used for industrial carbon fibers, but the temperature resistance of these agents is significantly lower than that of resin matrices based on high-temperature-resistant polyimide. In addition, the thermal stability and surface chemical characteristics of epoxy sizing agents often result in poor compatibility with certain resin-based composite molding processes. For example, typical thermoplastic composites require prepreg layup under high-temperature and aerobic conditions, which can substantially alter the chemical composition of epoxy sizing agents [42,43]. Likewise, epoxy sizing agents can have adverse effects on the molding process of thermosetting composites reinforced with polyimide. The presence of excess reactive epoxy groups in the sizing agents may lead to undesirable reactions with polyimide precursors, which function as curing agents. Early research on epoxy resin modification demonstrated that the use of polyamide acid as a curing agent for epoxy resin was an effective method to enhance bonding strength, thermal resistance, and mechanical performance [44,45,46]. However, the curing behavior of epoxy resin cured by polyamide acid is complex, involving self-imidization of the polyamide acid and competing reactions between polyamide acid and epoxy resin [47]. Similar behavior is observed during the molding of carbon-fiber composites coated with epoxy sizing, where the curing process of the polyimide-matrix resin is adversely affected. Destruction of the rigid cross-linked network leads to a reduction in the thermal resistance of the resin matrix. Therefore, further in-depth studies are necessary to fully understand the impact of epoxy sizing agents on the thermal resistance of polyimide-based composites.

This study investigates the influence of carbon-fiber sizing on the thermal properties of phenylacetylene-terminated polyimide (PEPI) composites. By analyzing curing kinetics and characterizing the chemical structure, the underlying mechanism by which epoxy-based sizing agents compromise the thermal resistance of polyimide composites was elucidated. Based on this mechanism, a strategy for tuning thermal resistance was developed and experimentally validated, offering a novel approach to enhancing the thermal performance of polyimide composites for engineering applications.

## 2. Materials and Methods

### 2.1. Materials

Phenylethynyl phthalic anhydride end-capped polyimide (PEPI) was developed by the Beijing Institute of Aeronautical Materials. Epoxy resin (E54) and hardener (D230) were obtained from Shanghai Huayi Resin Co., Ltd. (Shanghai, China) CCF800 carbon fiber was obtained from Weihai Expand Fiber Co., Ltd. (Weihai, China), and sized with E54/D230.

### 2.2. Blending of Epoxy–Polyimide Resin and Preparation of Molding Compounds

#### 2.2.1. Blending of Epoxy and Polyimide Resin

This study established four groups of blended resin experiments and two control groups. The specific blending ratios were provided in Table 1. The blending process followed a solution method, in which a predetermined amount of PEPI resin solution, E54, and D230 were added to a three-neck flask and stirred at room temperature for 4 h to form a blended resin solution. The solution was then poured out and subjected to stepwise heating treatment in a vacuum oven, with a maximum treatment temperature of 240 °C, maintained for 2 h. After treatment, a blended resin powder was obtained. The blended resins were labeled AE0 to AE3 and ADE according to the proportions of the components.

#### 2.2.2. Preparation of Blended Resin Molding Compounds

**Figure 1:**
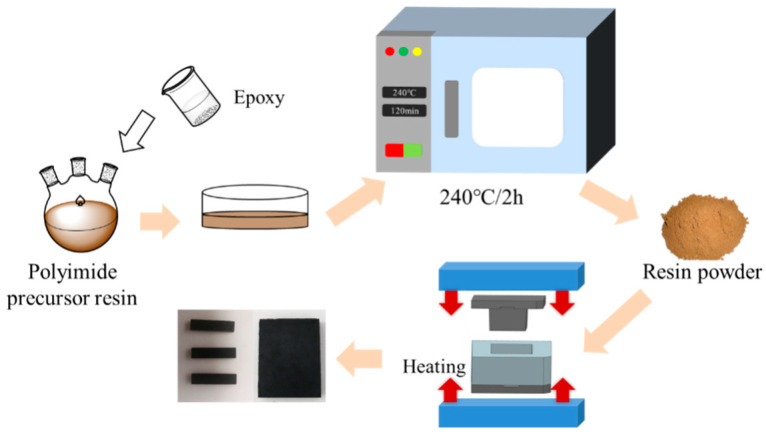
Schematic diagram of the preparation of blended resin molding compounds.

A total of 15 g of blended resin powder was poured into a mold. The mold was placed in a hot press and heated it until the mold temperature reached 350 °C. Pressure of 2.0 MPa was applied, and the temperature was maintained for 30 min. Then, the temperature was increased to 380 °C and held for 2.5 h. Afterward, the mold was cooled to 160 °C, the pressure was released, and the mold was removed to obtain the blended resin molding compound. The preparation process is shown in Figure 1.

### 2.3. Treatment of Carbon Fiber with Epoxy Sizing Agent and Preparation of Composites

#### 2.3.1. Treatment of Carbon Fiber with Epoxy Sizing Agent

**Figure 2:**
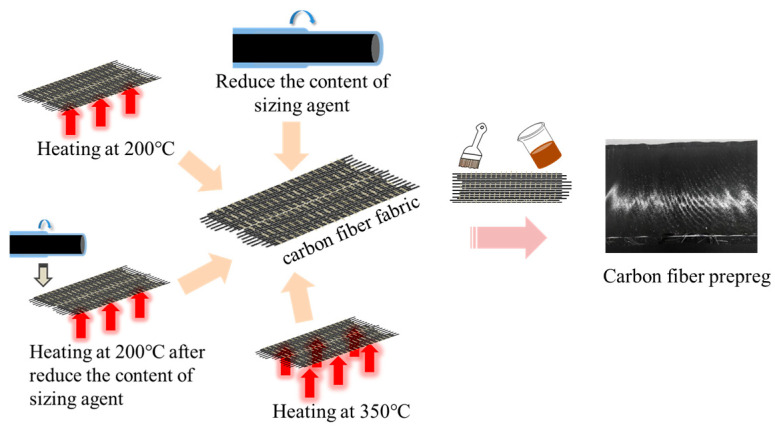
Schematic diagram of optimized treatment for epoxy-sized carbon fiber.

This study employed three treatment methods on epoxy-sized carbon fibers, as illustrated in Figure 2. (i) The content of the epoxy sizing agent on the carbon fibers was reduced from 1.0 wt.% to 0.5 wt.%, and the treated fibers were subsequently named CCF800-0.5ES. (ii) The epoxy-sized carbon fibers were subjected to heat treatment in a medium-temperature oven, using a stepwise heating approach. The maximum treatment temperature was set at 200 °C, maintained for 1 h. After treatment, the fibers were named CCF800-ESHT and CCF800-0.5ESHT, respectively. (iii) The epoxy-sized carbon fibers were placed in a high-temperature oven and heated to a maximum temperature of 350 °C, maintaining this temperature for 2 h. The fibers were then named CCF800-DS.

#### 2.3.2. Preparation of Polyimide Composite Laminates

The PEPI resin solution was manually applied to carbon-fiber scrim cloth to prepare pre-impregnated carbon-fiber material. The pre-impregnated material had a fiber areal density of 130 g/m^2^, with a resin content controlled at 35 ± 2%. The theoretical thickness of a single layer of the pre-impregnated material after curing was 0.125 mm. The pre-impregnated material was cut, laid out, vacuum-sealed, and placed in an oven for preheating at a maximum temperature of 200 °C for 2 h. After pretreatment, high-temperature auxiliaries were replaced, and the preform was vacuum-sealed again and placed in a hot press for curing. The maximum curing temperature was 380 °C, maintained for 2 h under a curing pressure of 2 MPa. After maintaining the temperature and pressure, the material was allowed to cool to below 100 °C before being removed from the press, resulting in a polyimide composite laminate.

### 2.4. Characterization

The thermal performance of the blended resin was evaluated using a differential scanning calorimeter (DSC250, TA Instruments, New Castle, DE, USA). Samples were heated from room temperature to 500 °C at heating rates of 2.5 °C/min, 5 °C/min, 10 °C/min, 15 °C/min, and 20 °C/min. The T_g_ of the resin and composites was determined using a dynamic mechanical analyzer (DMA 242E, NETZSCH, Selb, Germany). Under a nitrogen atmosphere, the samples were heated from room temperature to 450 °C at a heating rate of 5 °C/min. Resin samples were tested in a three-point bending fixture, and composite samples in a dual cantilever fixture, according to ASTM D7028 [48]. The thermal stability of the resin was assessed using a thermogravimetric analyzer (TGA550, TA Instruments, New Castle, DE, USA), where samples were heated from room temperature to 650 °C at a rate of 20 °C/min under a nitrogen atmosphere. Fourier transform infrared (FTIR) spectroscopy of the resin was recorded using a Thermo Fisher Scientific IS50 spectrometer (Waltham, MA, USA) to identify characteristic peaks of the resin structure based on the FTIR spectrum. The ^1^H-NMR spectrum of the blended resin was obtained at room temperature using a Bruker Fourier 300 instrument (Billerica, MA, USA), with dimethyl sulfoxide (DMSO) as the solvent. The surface of the carbon fibers and the internal morphology of the composites after failure were examined using a scanning electron microscope (Sigma 300, ZEISS, Oberkochen, Germany). X-ray photoelectron spectroscopy (XPS) analyses were performed using a VG Scientific ESCALab220i-XL instrument (West Sussex, UK) to observe changes in the chemical functional groups on the surface of the carbon fibers treated with epoxy sizing agents after adaptability optimization. Atomic force microscopy (AFM) analyses were conducted using a Bruker NanoIR3-AFM device (Billerica, MA, USA) to investigate the microstructure on the surface of the carbon fibers. The interlaminar shear strength (ILSS) of the composites was tested using an Instron-5567 universal testing machine (Norwood, MA, USA), characterizing the bond strength between the carbon fibers and the resin matrix according to ASTM D2344 [49]. The thermal aging weight loss of the composites was evaluated by wiping the surface of the composite strips with alcohol and heating them in a vacuum oven at 200 °C for 1 h to remove residual moisture. After this treatment, the weight of the strips was recorded as m_0_. The composite strips were then placed in a 400 °C oven for 100 h of isothermal thermal aging. During this period, the strips were periodically removed, cooled in a desiccator, and weighed. The thermal aging weight-loss rate of the composites was calculated using Formula (1), where w_t._ was the weight-loss rate (%), m_0_ was the weight before aging (g), and m_t_ was the weight after aging (g).

w_t_.% = (m_0_ − m_t_)/m_0_ × 100% (1)

## 3. Results and Discussion

### 3.1. Thermal Resistance of the Polyimide–Epoxy Compound

**Figure 3:**
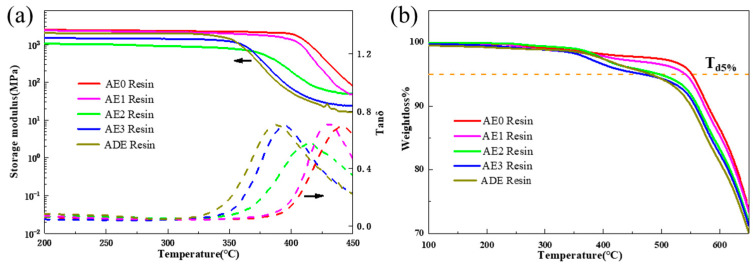
DMA curves (**a**) and TGA curves (**b**) of the blended resins.

Epoxy-based sizing agents are commonly applied to carbon fibers. During the composite molding process, these sizing agents diffuse into the resin matrix, forming a complex interfacial phase [37,38,39,50,51]. This study employed a blending method of epoxy and polyimide resins to simulate the internal behavior of epoxy sizing agents in polyimide composites. By analyzing the blended resin, the mechanism of epoxy sizing agents causing damage to the temperature resistance of polyimide composites was explored. The DMA curve of the blended resin is shown in Figure 3, clearly demonstrating a significant decrease in T_g_ after the addition of epoxy resin. Compared to the T_g_ of pure polyimide resin at 441 °C, the T_g_ of the blended resin (ADE), after incorporating 3.88% of E54/D230 epoxy resin, dropped to 385 °C, a reduction exceeding 50 °C. This phenomenon was similar to the substantial decrease in T_g_ observed in polyimide composites made with epoxy-sized carbon fibers, where the T_g_ of the composite also dropped by approximately 50 °C after blending with epoxy resin. As a low-temperature-resistant component, the blending of epoxy resin with high-temperature-resistant polyimide resin led to a reduction in the T_g_ due to the blending effect, which was calculated and assessed using the Fox–Flory equation [52], as shown in Equation (2):

T_g_ = (ω_1_∆C_p,1_T_g,1_ + ω_2_∆C_p,2_T_g,2_)/(ω_1_∆C_p,1_ + ω_2_∆C_p,2_) (2)

1/T_g_ = ω_1_/T_g,PI_ + ω_2_/T_g,EP_ (3)

**Figure 4:**
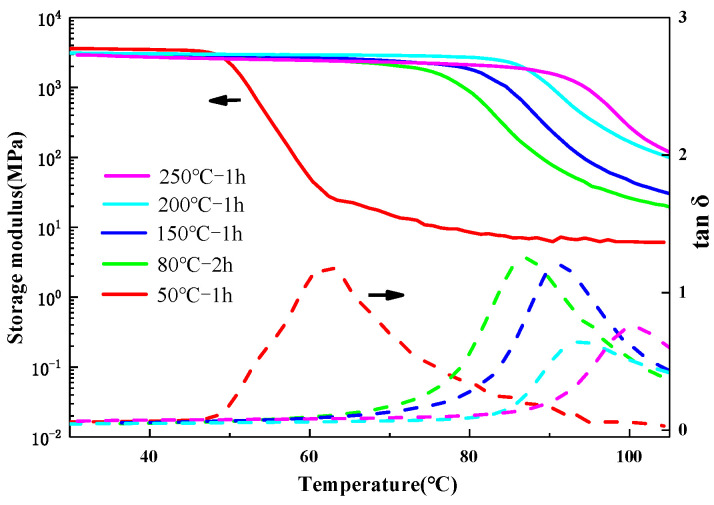
DMA curves of epoxy resin under different post-treatment conditions.

Assuming ∆C_p,i_T_g,i_ (i = 1, 2) is equal to a constant in the Fox–Flory equation, the T_g_ of the blended resin can be calculated using Formula (3). It was noted that the T_g_ of the epoxy resin might increase after blending with polyimide and undergoing high-temperature curing of the polyimide resin. To achieve a more accurate calculation, the epoxy resin strips were first post-cured in an oven, and the T_g_ of the post-cured strips was then incorporated into the formula. Figure 4 presents the DMA test curves of the epoxy resin under different post-treatment conditions. After a 1-h post-curing at 250 °C, the T_g_ of the epoxy resin reached 101 °C. As the post-treatment temperature increased, the rise in T_g_ became less significant, and further increases were avoided as the temperature approached the decomposition point of the resin. Specific values are listed in Table 2.

Calculations indicated that the theoretical T_g_ of the ADE blended resin was 417 °C, which still differed by approximately 30 °C from the actual T_g_ measured in tests. This discrepancy suggested that evaluating the impact of epoxy sizing agents on the temperature resistance of polyimide based solely on blending effects was inadequate. Furthermore, DMA and TGA test results of the blended resin demonstrated that as the proportion of epoxy resin increases, both the T_g_ and T_d5%_ of the blended resin decreased at a faster rate, highlighting the presence of other significant factors beyond blending effects.

### 3.2. Epoxy Influence on the Polyimide Curing Kinetics

To further investigate the factors affecting the temperature resistance degradation of polyimide composites with epoxy sizing agents, a kinetic analysis of the curing process was performed on blended resins (AE0~AE3). Common techniques for studying curing kinetics include Fourier transform infrared spectroscopy (FTIR), rheological analysis, dynamic mechanical analysis (DMA), and differential scanning calorimetry (DSC) [53,54,55,56]. Among these, DSC offers high precision and requires only small sample sizes; therefore, a non-isothermal DSC method was adopted to examine the curing kinetics of the blended resins.

**Figure 5:**
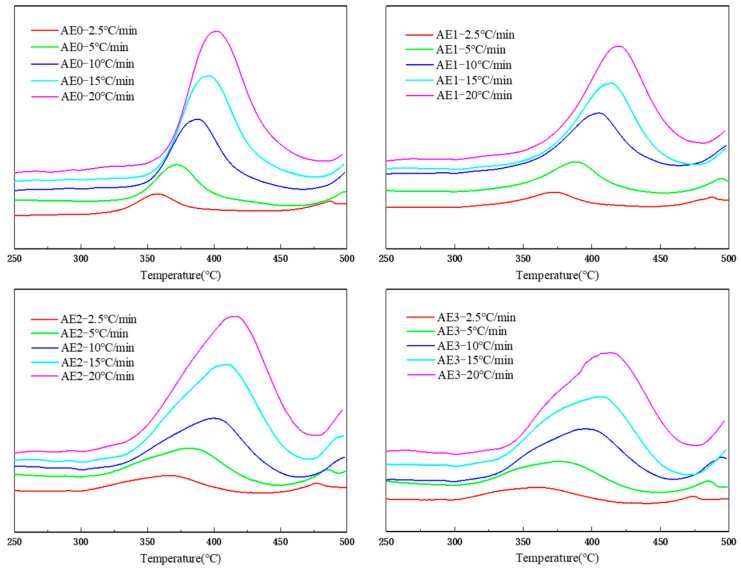
DSC curves of blended resins at different heating rates.

Figure 5 presents the DSC curves of the blended resins at heating rates of 2.5 °C/min, 5 °C/min, 10 °C/min, 15 °C/min, and 20 °C/min. The apparent activation energy of the blended resin was determined using the peak temperature (T_p_) of the exothermic curing peak, based on the Kissinger equation [57], as shown in Equation (4):

ln(β/T_P_^2^) = ln(AR/∆E) − ∆E/RT_P_ (4)

In the equation, ln(AR/∆E) represented a constant. By plotting the −ln(β/T_p_^2^) against 1/T_p_, the slope of the curve (∆E/R) could be used to calculate the activation energy of the reaction, ∆E. The pre-exponential factor A was then determined from the curve intercept ln(AR/∆E) and the previously calculated activation energy ∆E. The reaction order, n, was determined using the Crane equation [58], as shown in Equation (5):

d(lnβ)/d(1/T_P_) = −∆E/nR (5)

A linear relationship existed between lnβ and 1/T_p_ in the equation. The reaction order, n, was determined by calculating the slope of the lnβ versus 1/T_p_ curve, in combination with the previously calculated activation energy ∆E. The calculated values for the curing reaction activation energy (Ea), pre-exponential factor (A), and reaction order (n) of the blended resin were summarized in Table 3.

**Figure 6:**
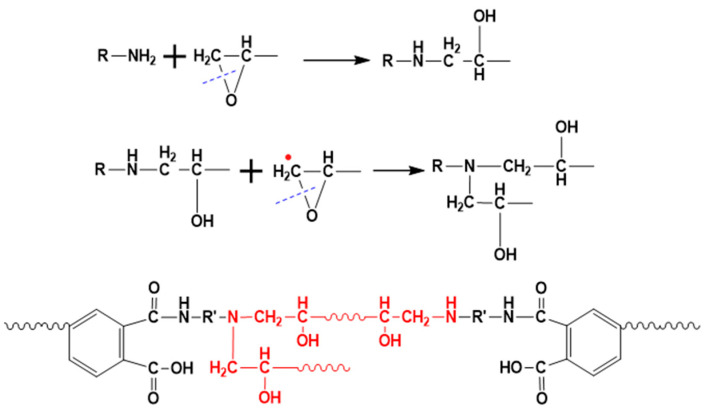
Possible reactions between epoxy groups and polyimide.

A comparison of the kinetic parameters of the curing process of blended resins revealed that epoxy resin has a significant influence on the curing behavior of the polyimide base resin. As the content of epoxy resin increases, the activation energy of the curing reaction of the blended resin gradually decreases. The curing process of phenylethynyl-terminated polyimide resin was generally considered to involve a crosslinking reaction between the terminal acetylene groups. A decrease in activation energy indicated that the curing reaction becomes easier and the resin oligomers possess stronger molecular mobility. Epoxy sizing containing a large number of epoxy groups will react with the diamines in the polyimide precursor before imidization, disrupting the original ratio of anhydride to amine in the polyimide. This will result in the incorporation of the epoxy resin molecular structure into the polyimide molecular chain, with the possible changes illustrated in Figure 6. The introduction of the epoxy resin structure will increase the flexibility of the polyimide main chain, promoting the likelihood of collisions between the terminal phenylethynyl groups and thereby facilitating the curing reaction. Simultaneously, the lengthening of the molecular chains will reduce the density of the three-dimensional network formed during curing, leading to a decrease in the T_g_ of the cured resin.

### 3.3. Structure Characterization of the Polyimide–Epoxy Compound

**Figure 7:**
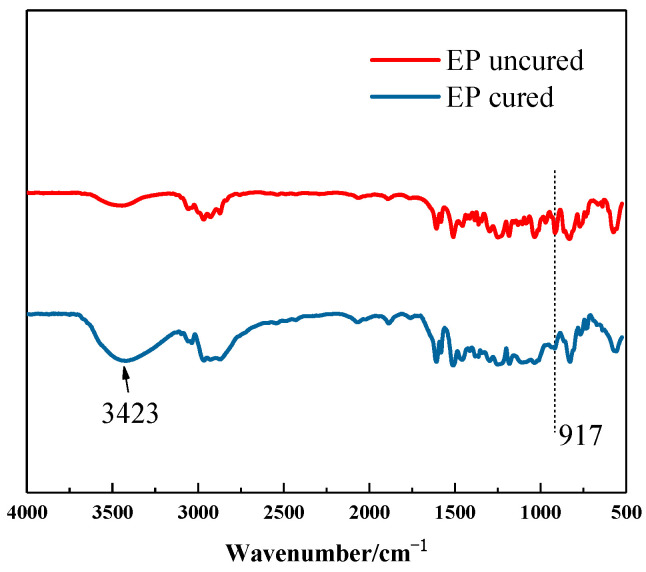
Infrared spectra of epoxy resin before and after curing.

**Figure 8:**
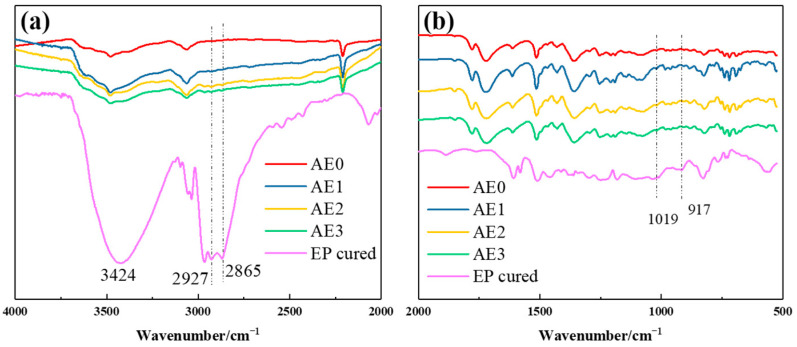
Infrared spectra of blended resin (**a**) 4000 cm^−1^ to 2000 cm^−1^ (**b**) 2000 cm^−1^ to 500 cm^−1^.

To verify the aforementioned hypothesis, infrared spectroscopy analysis was performed on the blended resin. Epoxy groups in epoxy resin exhibited high thermal stability and do not undergo ring-opening reactions in the absence of a curing agent. However, when reactive amino groups were introduced into the system, they interact with the epoxy groups, inducing ring-opening. Figure 7 presents the infrared spectra of the epoxy resin before and after curing. The uncured epoxy resin showed a distinct absorption peak at 917 cm^−1^, which almost disappears after curing with amino groups. A broad absorption peak appeared around 3400 cm^−1^, likely due to the formation of hydroxyl groups from the ring-opening of epoxy groups. In the infrared spectra of the blended resin (Figure 8), no distinct peaks associated with epoxy groups were detected. A weak absorption peak corresponding to the -N-CH_2_- structure was detected at 1019 cm^−1^, while characteristic peaks of -CH_2_ and -CH_3_ from the epoxy main chain appeared at 2927 cm^−1^ and 2865 cm^−1^, respectively. Additionally, the absorption peaks in the 3400–3600 cm^−1^ range were noticeably broader.

**Figure 9:**
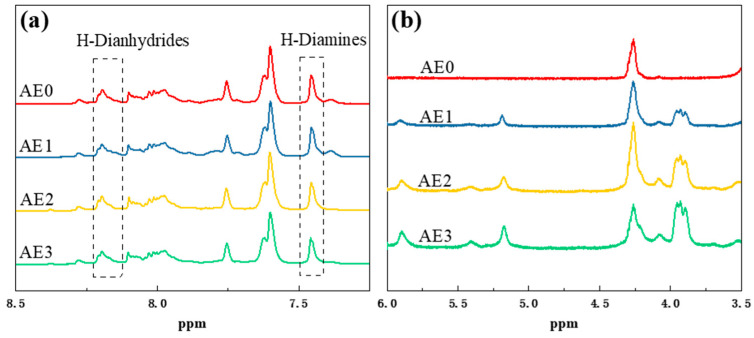
^1^H-NMR spectra of blended resin (**a**) 8.5 ppm to 7.25 ppm (**b**) 6.0 ppm to 3.5 ppm.

Further analysis of the blended resin was performed using proton nuclear magnetic resonance spectroscopy, as shown in Figure 9. Active hydrogen absorption peaks were observed near the 4 ppm chemical shift, with peak areas increasing as the epoxy resin content increased. This was likely attributed to the formation of -OH and -NH- groups resulting from the ring-opening of the epoxy groups. At chemical shifts of 7.45 ppm and 8.2 ppm, hydrogen attached to the aromatic diamines and dianhydrides within the molecular chain was identified. Integration of the peak areas indicated a gradual decrease in the proportion of anhydride-related hydrogen in the polyimide main chain after the introduction of epoxy resin. This suggested that the epoxy groups preferentially reacted with the aromatic diamines and dianhydrides, preventing the usual binding between the dianhydrides and diamines and leading to the exclusion of some aromatic dianhydrides from the polyimide main chain. Infrared spectroscopy and nuclear magnetic hydrogen spectroscopy analyses of the blended resin confirmed the hypothesis that the epoxy groups undergo ring-opening reactions with the diamines in the polyimide precursor.

Epoxy sizing agents are commonly applied to carbon fibers; however, the ratio of epoxy resin to curing agent in these sizing formulations is intentionally nonequimolar. To retain sufficient flexibility after sizing, only a small amount of curing agent was incorporated, leaving a large number of unreacted epoxy groups on the carbon-fiber surface. During the fabrication of PEPI composites, the polyimide precursor solution was typically combined with the epoxy-sized fibers to produce prepregs. These prepregs were subsequently heated to achieve imidization of the PEPI resin, followed by high-temperature, high-pressure curing to complete composite consolidation. Based on studies of blended epoxy–PEPI systems, it was speculated that the surface-bound epoxy groups on epoxy-sized fibers could react with the diamine components of the PEPI matrix during processing. These reactions may allow epoxy species to gradually diffuse from the fiber-matrix interphase into the bulk matrix, thereby disturbing the designed stoichiometric ratio between dianhydride and diamine in the PEPI main chain. This imbalance promoted the formation of a lower-temperature cross-linked structure, ultimately reducing the thermal resistance of PEPI composites.

### 3.4. Strategy to Enhance the Thermal Resistance

**Figure 10:**
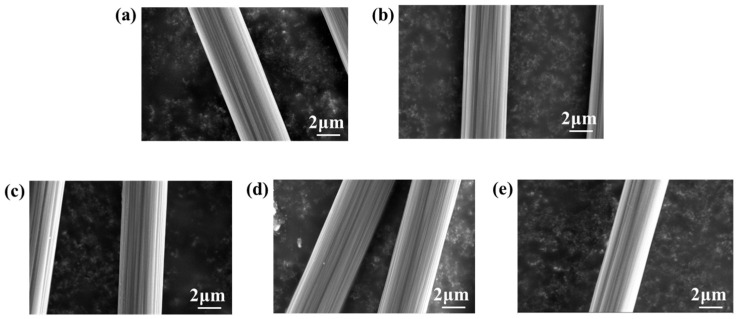
SEM images of (**a**) CCF800-ES, (**b**) CCF800-0.5ES, (**c**) CCF800-ESHT, (**d**) CCF800-0.5ESHT and (**e**) CCF800-DS.

**Figure 11:**
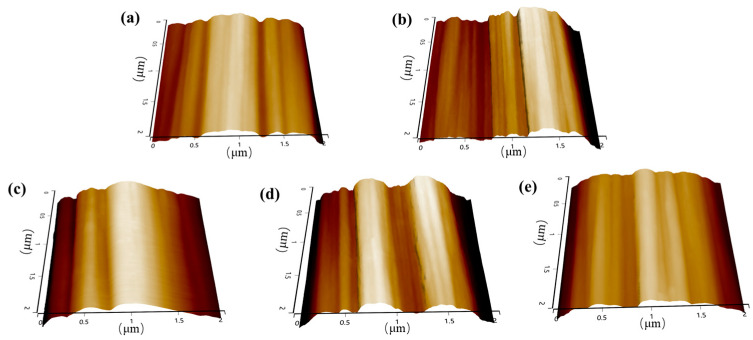
AFM images of (**a**) CCF800-ES, (**b**) CCF800-0.5ES, (**c**) CCF800-ESHT, (**d**) CCF800-0.5ESHT and (**e**) CCF800-DS.

**Figure 12:**
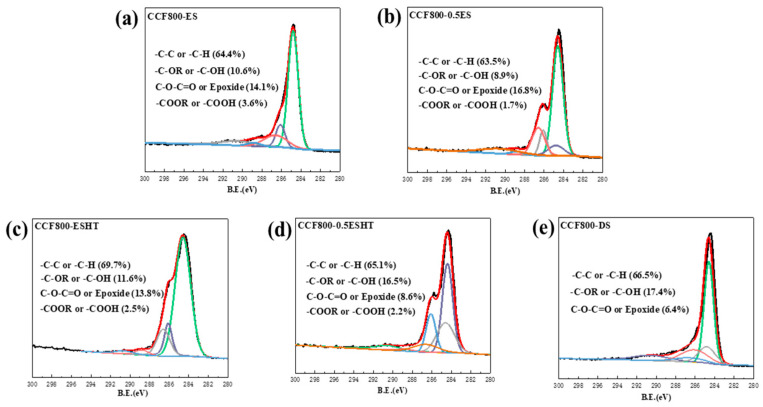
C1s peak spectra of (**a**) CCF800-ES, (**b**) CCF800-0.5ES, (**c**) CCF800-ESHT, (**d**) CCF800-0.5ESHT and (**e**) CCF800-DS.

**Figure 13:**
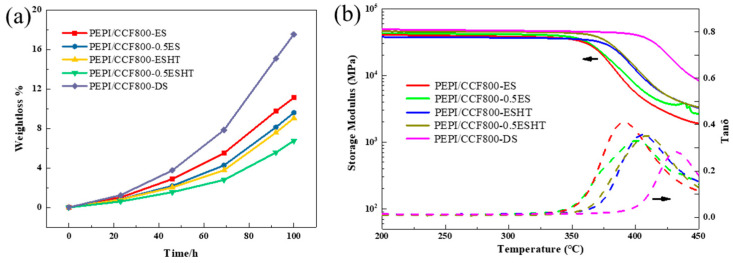
Composites thermal aging weight-loss curves (**a**) and DMA Test Curves (**b**).

To mitigate the adverse effects of epoxy sizing agents on the thermal resistance of PEPI composites and minimize the impact of blending, while ensuring the bundling of carbon fibers, the content of epoxy sizing agents on the fibers was reduced. After decreasing the epoxy sizing agent content, no significant changes were observed in the surface morphology of the carbon fibers, as shown in Figure 10. In the wet spinning process, fiber surface collapse may occur due to the double diffusion effects. Characterization results showed that the epoxy sizing agent did not significantly alter the groove-like surface morphology of the carbon fibers. Even after various subsequent treatments, the fibers retained abundant grooves, as illustrated in Figure 11. Additionally, no significant changes in the surface functional groups of the carbon fibers were detected (Figure 12). PEPI composite laminates were then prepared using carbon fibers with reduced epoxy sizing agent content. Tests revealed that the T_g_ of the PEPI/CCF800-0.5ES composite material was 401 °C, showing an improvement of approximately 10 °C compared to previous results (Figure 13b). Moreover, the thermal aging performance of the composites was enhanced. After 100 h of thermal aging at 400 °C, the weight-loss rates of PEPI/CCF800-ES and PEPI/CCF800-0.5ES were 11.11% and 9.59%, respectively (Figure 13a). Reducing the content of the epoxy sizing agent slightly improved the overall thermal resistance of the polyimide composites.

**Figure 14:**
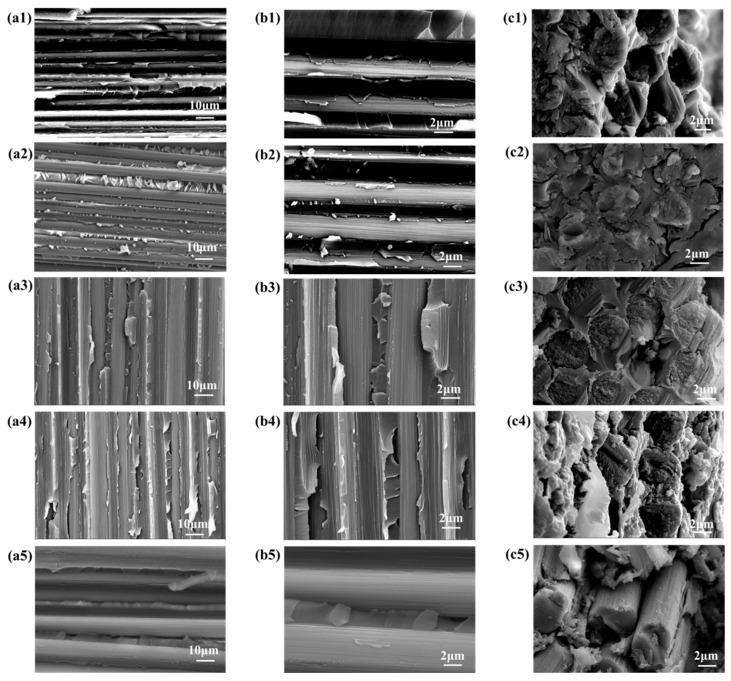
Typical SEM morphology of polyimide composites. (**a1**,**b1**,**c1**) for PEPI/CCF800-ES; (**a2**,**b2**,**c2**) for PEPI/CCF800-0.5ES; (**a3**,**b3**,**c3**) for PEPI/CCF800-ESHT; (**a4**,**b4**,**c4**) for PEPI/CCF800-0.5ESHT; (**a5**,**b5**,**c5**) for PEPI/CCF800-DS.

To further mitigate the detrimental effects of active epoxy groups in epoxy sizing agents on polyimide, the epoxy-sized carbon fibers were subjected to heat treatment in an oven to facilitate the ring-opening deactivation of the epoxy groups on the fiber surfaces. Following this heat treatment, the number of epoxy groups on the fiber surfaces decreased, while the content of hydroxyl groups increased. However, the total number of surface-active functional groups remained unchanged, as shown in Figure 12. In terms of flexibility, CCF800-ESHT became slightly more rigid, while CCF800-0.5ESHT maintained flexibility similar to that of CCF800-ES. Furthermore, polyimide composite laminates were fabricated using the treated carbon fibers. Testing revealed a significant improvement in the temperature resistance of the composites following medium-temperature pretreatment. The T_g_ of PEPI/CCF800-0.5ESHT reached 410 °C, an increase of approximately 20 °C compared to PEPI/CCF800-ES. Simultaneously, the thermal aging weight loss was significantly reduced, with PEPI/CCF800-ESHT and PEPI/CCF800-0.5ESHT exhibiting weight-loss rates of 9.04% and 6.75%, respectively, after 100 h of thermal aging at 400 °C. Moreover, the composite exhibited an interlayer shear strength (ILSS) of 112 MPa, and after thermal aging at 400 °C for 100 h, the ILSS remained at 77 MPa, demonstrating excellent strength retention. Post-failure analysis of the internal surface and cross-sectional morphology indicated that the carbon fibers were still firmly bonded to the resin matrix, as shown in Figure 14.

To eliminate the negative impact of epoxy sizing agents on the thermal resistance of polyimide composites and to achieve the deactivation and decomposition of the epoxy sizing agent on the carbon-fiber surfaces, the epoxy-sized carbon fibers were subjected to high-temperature heating in an oven. After treatment, the epoxy group content on the carbon-fiber surfaces significantly decreased, and the total number of active functional groups dropped from 28.3% to 23.8%, as shown in Figure 12e. At the same time, the fibers lost their bundling, and visible dispersion within the fiber bundles was observed. Polyimide composite laminates were subsequently prepared using the treated carbon fibers. Tests showed that the T_g_ of PEPI/CCF800-DS reached 432 °C, an increase of approximately 40 °C compared to PEPI/CCF800-ES, approaching the T_g_ of the bulk resin. However, in the thermal aging tests, PEPI/CCF800-DS exhibited a weight loss of 17.54% after 100 h at 400 °C, significantly higher than that of PEPI/CCF800-ES. Additionally, the ILSS of PEPI/CCF800-DS decreased by approximately 20%. Further observations of the surface and cross-sectional morphology of the composites revealed extensive exposure of carbon fibers at the break points, with minimal resin encapsulation. The matrix resin between fibers showed brittle fracture, and the fracture surfaces were smooth, as shown in Figure 14(a5,b5). The presence of distinct fiber pull-out features in the composite fracture morphology, as shown in Figure 14(c5), indicated that the internal carbon fibers and the resin matrix might detach when subjected to external forces. High-temperature de-sizing treatment could deactivate and decompose the epoxy sizing agent on the carbon-fiber surfaces, significantly increasing the T_g_ of the composites; however, it negatively affected the interface between the carbon fibers and the resin matrix, leading to reduced thermal resistance and bond strength at the composite interface.

**Figure 15:**
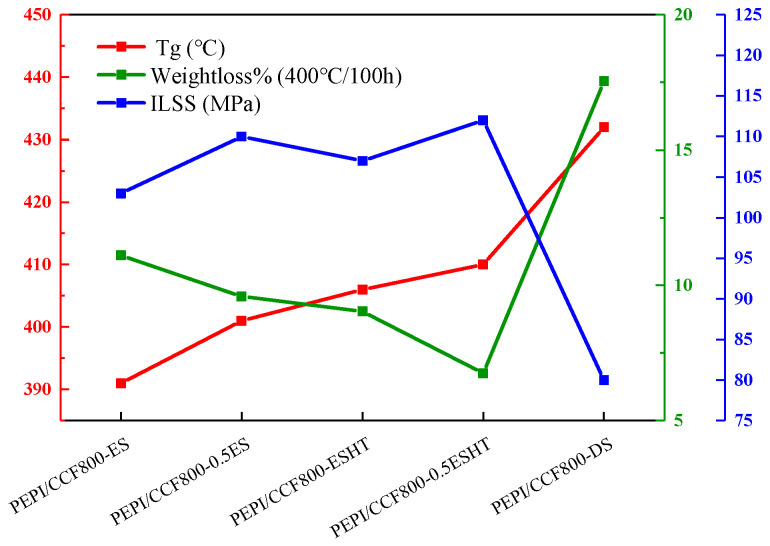
The summary results of polyimide composites.

The overall findings were summarized in Figure 15. Heat-treating and precuring epoxy-sized fibers effectively enhanced the thermal resistance of PEPI composites. Compared with conventional epoxy-sized systems, the T_g_ increased from 391 °C to 410 °C, and the weight-loss rate after 100 h of thermal aging at 400 °C decreased from 11.11% to 6.75%, indicating a remarkable improvement in thermal stability. The ILSS of the PEPI composite reached 112 MPa, comparable to values reported for PEPI composites prepared using polyimide-sized fibers [59]. For polyimide-matrix composites, the use of polyimide-based sizing agents remains an important research direction. However, polyimide sizing agents often cause excessive stiffening of the fibers, leading to fuzzing and processing difficulties; as a result, their industrial application requires intricate preparation and post-treatment procedures. In contrast, epoxy sizing agents are already widely used in industry and offer mature, stable processing routes. The heat treatment applied to epoxy-sized fibers in this study significantly improved fiber–PEPI-matrix compatibility and provided a simple, scalable, and industry-ready approach for enhancing the performance of PEPI composites.

## 4. Conclusions

The influence of carbon-fiber sizing on the thermal properties of phenylethynyl -terminated polyimide (PEPI) composite was studied, based on which a strategy was established to enhance the thermal resistance. The epoxy sizing exhibited a nonlinear detriment to the PEPI thermal resistance, suggested by the deviation of the glass transition temperature (T_g_) of PEPI from the linear Fox–Flory equation calculation by 26 °C. This nonlinear detriment effect may be ascribed to the thermal curing mechanism change, possibly arising from the insertion of a flexible segment into the polyimide chain by epoxy–amine reaction. A corresponding strategy to manipulate the thermal resistance was established and verified by carbon-fiber precuring. By fiber precuring at 200 °C, the PEPI composite exhibited a T_g_ enhancement by 20 °C, and a 100 h weight-loss reduction under thermal aging at 400 °C from 11.11% to 6.75%. Simultaneously, a good fiber-matrix interface was maintained, as suggested by a short beam shear strength higher than 100 MPa. This strategy provides a new route to enhance the thermal properties of polyimide composites from the perspective of improving the carbon fiber-matrix compatibility, which has significant engineering application potential.

## Figures and Tables

**Table 1:** Blending ratios of resins.

Sample	AE0	AE1	AE2	AE3	ADE	Epoxy
PEPI/g	44.592	44.592	44.592	44.592	44.592	0
E54/g	0	0.43	1.37	1.8	1.37	1.37
D230/g	0	0	0	0	0.43	0.43
Epoxy/(PI + Epoxy)	0	0.955%	2.98%	3.88%	3.88%	1

**Table 2:** Calculated T_g_ of blended resins.

Sample	T_g_ (Tanδ) Actual Measurement	T_g_ (Tanδ) Theoretical Calculation
PEPI resin	441 °C	441 °C
E54/D230	101 °C	101 °C
ADE resin	385 °C	417 °C
PEPI/CCF800 composite	391 °C	417 °C

**Table 3:** Kinetic parameters of curing reaction for AE series blended resins.

Sample	Ea [kJ/mol]	lnA	n
AE0	150.74	15.58	0.9329
AE1	150.08	14.70	0.9310
AE2	141.68	13.38	0.9278
AE3	132.96	11.90	0.9238

## Data Availability

The original contributions presented in this study are included in the article. Further inquiries can be directed to the corresponding author.
